# Evaluation of Histological Criteria and Immunoserological Testing of Simplified Criteria for the Diagnosis of Autoimmune Hepatitis

**DOI:** 10.5152/tjg.2025.25402

**Published:** 2025-09-18

**Authors:** Kenan Moral, Cumali Efe, Ayşenur Sert, Berkay Şimşek, Dilara Turan Gökçe, Nergiz Ekmen, Ersin Batıbay, Murat Kekilli, Tarkan Karakan, Mehmet Ibiş, Güner Kiliç, Ersan Ozaslan, Haluk Cihad Albayrak, Veysel Baran Tomar, Derya Arı, Dilek Yapar, Meral Akdoğan, Mehmet Cindoruk, Staffan Wahlin, Guldal Esendagli, Nesrin Turhan, Gulen Akyol

**Affiliations:** 1 Department of Gastroenterology and Hepatology, Gazi University School of Medicine, Ankara, Türkiye; 2 Department of Gastroenterology, Harran University Hospital, Şanlıurfa, Türkiye; 3 Department of Pathology, Gazi University School of Medicine, Ankara, Türkiye; 4 Department of Gastroenterology, Sincan State Hospital, Ankara, Türkiye; 5 Department of Gastroenterology, Ankara Bilkent City Hospital, University of Health Sciences, Ankara, Türkiye; 6 Department of Internal Medicine, Gazi University School of Medicine, Ankara, Türkiye; 7 Institute of Health Science, Department of Medical Informatics, Akdeniz University, Antalya, Türkiye; 8 Division of Hepatology, Department of Upper GI Diseases, Karolinska Institutet, Karolinska University Hospital, Stockholm, Sweden; 9 Department of Pathology, University of Health Sciences, Ankara Bilkent City Hospital, Ankara, Türkiye

**Keywords:** Liver cirrhosis, liver failure, liver transplantation, mercaptopurine, rituximab

## Abstract

**Background/Aims:**

The International Autoimmune Hepatitis (AIH) Group recommends the new histological criteria (HC) (2022) and modified immunoserological testing for diagnosing AIH. The diagnostic utility of the 2022 HC was evaluated. The simplified criteria were also updated with the 2022 HC and immunoserological testing and assessed the diagnostic performance.

**Materials and Methods:**

The data of 207 patients (111 AIH, 33 primary biliary cholangitis, 35 drug-induced liver injury, and 28 metabolic dysfunction–associated fatty liver disease) were evaluated.

**Results:**

The 2022 HC and the 2008 simplified HC showed 95% vs. 88% sensitivity and 82% vs. 49% specificity for possible/compatible AIH. For likely/typical AIH, sensitivity was 60% vs. 42% and specificity was 98% vs. 95% for the 2022 HC and the 2008 HC, respectively. The area under the curve (AUC) was better for the 2022 HC than for the 2008 simplified HC (0.932 vs. 0.771, *P* < .001). The updated simplified criteria had a sensitivity comparable with the simplified criteria (88% vs. 87%) but a better specificity (94% vs. 80%) for probable AIH. The sensitivity was slightly lower (57% vs. 63%), but the specificity was greater (97% vs. 89%) for definitive AIH. The AUC was higher in the updated simplified criteria than in the simplified criteria (0.959 vs. 0.894, *P* = .016).

**Conclusion:**

The 2022 HC showed better sensitivity and specificity than the 2008 simplified HC for AIH. The updated simplified criteria worked well with improved accuracy of AIH diagnosis. Our results suggest that the diagnostic algorithm of AIH should be modified based on recent recommendations.

**Main Points**

- The International Autoimmune Hepatitis (AIH) Group recommends new histological criteria (HC) (2022) and modified immunoserological testing for diagnosing AIH.
- The simplified criteria were updated with the 2022 HC and immunoserological testing.
- The 2022 HC showed better sensitivity and specificity than the 2008 simplified HC.
- The updated simplified criteria improved the accuracy of AIH diagnosis.
- The diagnostic algorithm of AIH should be modified based on recent recommendations.

## Introduction

Autoimmune hepatitis (AIH) is a rare cause of liver disorder with unknown etiology. Autoimmune hepatitis often progresses into liver failure, cirrhosis, and death if untreated.^1^^-^^4^ Early recognition and prompt immunosuppressive therapy are lifesaving.

Autoimmune hepatitis is diagnosed based on a combination of laboratory and liver histology findings and exclusion of other causes of liver injury.^1^^-^^3^ To standardize the diagnostic algorithm for AIH, the International Autoimmune Hepatitis Group (IAIHG) proposed a revised scoring system in 1999, which includes 12 diagnostic parameters and is very complex to use in clinical practice.^5^ In 2008, the IAIHG proposed simplified criteria that include only 4 variables: autoantibodies, serum immunoglobulin (IgG) level, result of chronic viral hepatitis testing, and liver histology findings.^6^ According to these simplified criteria, each variable is attributed 0-2 points. A cumulative score of 6 is considered “probable AIH,” and a score of 7 is “definite AIH.”

Liver histology is important for establishing an AIH diagnosis, especially in individuals presenting without classical serological features and normal serum IgG levels. According to the simplified criteria,^6^ portal lymphocytic/lymphoplasmacytic infiltrates extending into the lobules, emperipolesis, and hepatic rosette formation are typical biopsy features. Few studies have evaluated the predictive performance of histological features in the simplified HC.^7^^,^^8^ In 1 study,^7^ emperipolesis and rosette formation were found as diagnostic hallmark features of AIH, while another study^8^ reported that emperipolesis and rosettes are difficult to interpret without establishing optimal sensitivity and specificity for AIH diagnosis. In 2022, the international AIH Pathology Group (AIH-PG) proposed a new HC that categorizes histological features as likely, possible, or unlikely manifestations of AIH.^9^ To date, only a study from China has validated the diagnostic accuracy of the AIH-PG criteria by using a sizable patient cohort. In this study,^10^ the new HC showed significantly better diagnostic performance than the old simplified HC. More data about the diagnostic utility of the new AIH-PG criteria are needed before they can be incorporated into routine care or prospective studies.

In 2021, the IAIHG recommended a modified methodology of immunoserological testing for AIH to better define reference values.^11^ The cutoff for a positive value of antinuclear autoantibodies (ANA) was increased when assessed on human epithelioma (HEp-2) cells. This management is expected to improve the specificity of the scoring systems for AIH. External validation of the new serological criteria is, however, needed.

The aim was to validate the diagnostic utility of the new histologic criteria and the updated simplified criteria that incorporate both the new histologic criteria and the modified methodology of immunoserological testing into the simplified criteria. Data from a sizeable cohort of patients with liver disease were collected and re-analyzed.

## Materials and Methods

### Data Collection

We retrospectively evaluated the data of patients who were diagnosed with AIH, primary biliary cirrhosis (PBC), drug-induced liver injury (DILI), and metabolic dysfunction–associated fatty liver disease (MAFLD) in 3 centers from Türkiye between January 2013 and March 2021. All study collaborators independently identified cases and collected patient data that included general information of patients, laboratory values, and autoimmune serology at the time of diagnosis. The IAIHG simplified score was re-calculated from collected data. All patients with AIH, PBC, MAFLD, and DILI had been diagnosed and treated according to international guidelines.^1^^,^^12^^-^^14^ Acute AIH was defined as transaminases higher than 10 times the upper normal limit with jaundice (total bilirubin > 3 mg/dL) at presentation.^15^ Elevated transaminases with prolonged INR > 1.5 and presence of hepatic encephalopathy were defined as acute AIH with liver failure.^16^ Drug-induced liver injury was considered in the presence of a history of drug ingestion within 6 months of onset of illness and exclusion of other common causes of liver injury (viral hepatitis and significant alcohol intake) in accordance with the suggested diagnostic approach.^13^ In cases with features of AIH, short-term steroid therapy with successful subsequent treatment withdrawal confirmed the DILI diagnosis. The study protocol was approved by the ethical committee of Ankara Bilkent City Hospital (Approval no: 2-24-438, Date: September 4, 2024). Informed consent was waived due to the retrospective nature of this study.

### Serological Evaluation

Immunofluorescence (IF) assay was performed for the detection of ANA (Mosaic Hep-20-10/primate liver), smooth muscle antibodies (rat, stomach), liver kidney microsomal type 1 (LKM1) antibodies, and anti-mitochondrial antibodies (AMA) (LKM+ mitocondria/rat, kidney) by using commercially available (Euroimmun, Lubeck, Germany) kits. The immunoblotting technique was used for the detection of soluble liver antigen/liver pancreas (SLA/LP) antibodies, anti-LKM-1, and AMA with the M2 fraction by using commercially available (Euroimmun, Lubeck, Germany) kits. For the IF assay, a titer of 1/40 or higher was considered positive for ANA, SMA, LKM-1, and AMA. A titer of 1/160 was considered positive when HEp-2 cells subtract was used for ANA detection.^11^

### Histopathological Evaluation

Re-evaluation of liver biopsies stained with hematoxylin and eosin, Masson’s trichrome, and methyl green pyronin was conducted. Histochemical stains, including rhodanin, periodic-acid Schiff, periodic-acid Schiff with diastase, Congo red, and gentian violet, as well as immunohistochemical stains, such as cytokeratin 19, IgG, and IgG4, were applied if necessary for the purpose of differential diagnosis. Semi-quantitative evaluation of plasma cells was performed. A plasma cell cluster was defined as the presence of ≥5 plasma cells in any foci of the portal and/or lobular areas. The inflammatory activity was characterized using Ishak’s modified histological activity index (mHAI). Additionally, Ishak staging was employed to assess fibrosis score 0-6.^17^ All liver biopsy findings were evaluated by 2 expert hepato-pathologists in a double-blind way, and both pathologists only evaluated their own institutional data.

### Autoimmune Hepatitis Histological Criteria

All cases were assessed according to the histological features suggested by both the old simplified HC^6^ and the new AIH-PG criteria.^9^ Autoimmune hepatitis was categorized as “typical” (score = 2), “compatible” (score = 1), or “atypical” (score = 0) in accordance with the old HC.^6^ The new 2022 AIH-PG initially categorized histologic features as either portal (chronic) or lobular (acute) hepatitis based on the predominant site of inflammation.^9^ Biopsies with either portal or lobular hepatitis were classified as “likely” (score = 2), “possible” (score = 1), or “unlikely” (score = 0). The new histological features are presented with details in Table 1.

### Updated Simplified Criteria in Autoimmune Hepatitis

For the main purpose of this study, serological parameters were updated according to the new adjusted IAIHG cutoff values.^11^ Also, the new histology criteria were incorporated into the simplified criteria (Table 2). Scores derived from these new, updated, simplified criteria were calculated. A score of ≥6 was considered probable AIH, whereas a score of ≥7 was considered definite AIH. The performance of both the simplified criteria and the updated simplified criteria for probable and definitive AIH was then evaluated.

### Statistical Analysis

Data analysis was performed using IBM SPSS version 26.0 (IBM SPSS Corp.; Armonk, NY, USA) and Phytoon. Categorical variables are presented as numbers and percentages, whereas normally distributed variables are expressed as mean with SD. The median (minimum-maximum) and range of 25%-75% are presented for non-normally distributed variables. Sensitivity, specificity, and predictability for each scoring system were calculated. The diagnostic values of both scoring systems were assessed by the area under the receiver operating characteristic curve. Chi-square was employed for comparing intergroup categorical variables, while the McNemar test was used to determine the difference in the dichotomous dependent variables. The comparison of receiver operating characteristic curves was performed by using the DeLong test. A *P* value < .05 was considered statistically significant.

## Results

### Patient Characteristics

We included 207 patients (75% female) with liver disease. Among them, 111 were diagnosed with AIH (85% female, median age at diagnosis: 53 years), 33 with PBC (82% female, median age at diagnosis: 53 years), 35 with DILI (63% female, median age at diagnosis: 46 years), and 28 with MAFLD (46% female, median age of diagnosis: 50). Median follow-up duration was 6 years for patients with AIH, 5 years for PBC, 6 months for DILI cases and 4 years for MAFLD patients. The preliminary data from 78 of these patients have already been presented in our previous study.^18^ A total of 35 patients with AIH demonstrated an acute presentation at the time of diagnosis. Anti-mitochondrial antibodies were not detected in 2 of the PBC patients, while 1 had PBC-specific ANA (multiple nuclear dots pattern) with a titer of 1/320. Six of the DILI cases who showed features of AIH at the time of presentation were given corticosteroid therapy for 3-4 months, and no relapse was observed after therapy withdrawal. Table 3 shows the general characteristics and laboratory features of the study population.

### Histopathological Features

The results of histological analysis of the study population are summarized in Table 4. In the AIH group, interface hepatitis was noted in 95%, portal lymphoplasmacytic infiltrate in 88%, lobular lymphoplasmacytic infiltrate in 41%, rosette formation in 68% and emperipolesis in 59%. In the PBC group, 76% patients showed interface hepatitis, 100% portal lymphoplasmacytic infiltration, 24% lobular lymphoplasmacytic infiltration, and 3% demonstrated rosette formation. In the DILI group, interface hepatitis was seen in 46%, portal lymphoplasmacytic infiltration in 83%, lobular lymphoplasmacytic infiltration in 26%, rosette formation in 23%, and emperipolesis in 11%. In the MAFLD group, interface hepatitis was seen in 11%, portal lymphoplasmacytic infiltration in 35%, lobular lymphoplasmacytic infiltration in 7%, rosette formation in 0%, and emperipolesis in 3%.

### Application of Histological Scoring Systems

**Figure 1.**
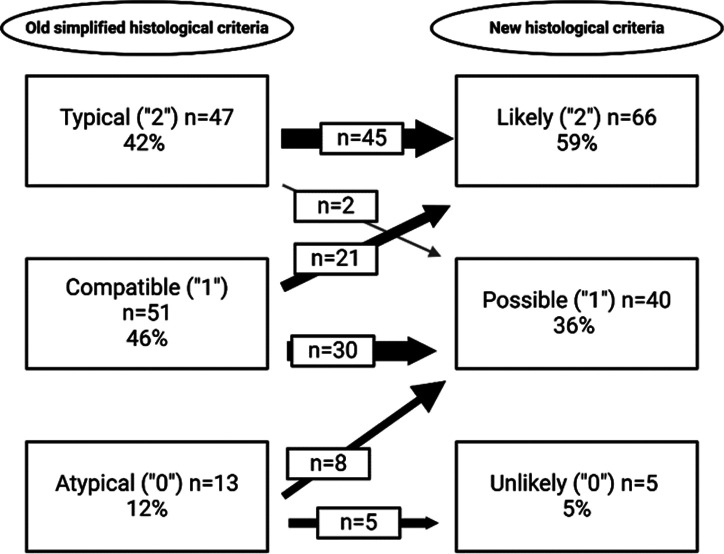
Comparison and transitions between the simplified histological criteria and the new histological criteria in the autoimmune hepatitis group.

The old HC classified 42 % (n = 47) as typical, 45% (n = 51) as compatible, and 11% (n = 13) of patients as atypical AIH. The new HC identified 59% (n = 66) as likely, 36 % (n = 40) as possible, and 4 % (n = 5) as unlikely AIH. The new HC upgraded 21 (19 %) patients from compatible to likely AIH, and 8 (7%) from atypical to compatible, while 2 (1.8 %) patients were downgraded from typical to possible AIH. The difference and transition of AIH patients according to the 2 different HC are presented in Figure 1.

Among 35 DILI patients, 4 (11%) were classified as typical AIH, 12 (34%) as compatible AIH, and 19 (54%) as atypical AIH according to the old HC. The new HC defined 2 (5%) as likely, 9 (25%) as possible, and 24 (68%) as unlikely AIH. The new HC downgraded 11 DILI patients (2 from typical to possible and 9 from compatible to unlikely) while 4 DILI patients were upgraded from atypical to possible AIH (Supplementary Figure 1).

Among 33 PBC patents, 29 (88%) patients were classified as compatible and 4 (12%) as atypical AIH according to the old HC. The new HC defined 6 (18%) patients as possible and 27 (82%) as unlikely AIH. The 2022 criteria downgraded 23 PBC patients from compatible to unlikely AIH (Supplementary Figure 2).

According to the old HC, 25 of 28 MAFLD patients were defined as atypical and 3 patients as compatible with AIH, while the new HC designated all 28 MAFLD patients as atypical AIH (Supplementary Figure 3).

Overall, the new HC showed 95% sensitivity and 82% specificity for possible AIH, while sensitivity was 60% and specificity was 98% for likely AIH (Table 5). The positive predictive value (PPV) and the negative predictive value (NPV) were 86% and 94% for possible AIH, 97% and 68% for likely AIH.

The sensitivity of the old HC was 88% for compatible AIH and 42% for typical AIH. The specificity was 49% and 95% for compatible and typical AIH, respectively. The PPV and NPV were 67% and 78% for compatible AIH, 90% and 59% for typical AIH.

### Performance of Updated Simplified Criteria

We updated the simplified criteria by incorporating the new serological testing and the new HC (Table 2). The same cutoffs were used in the updated simplified criteria as in the simplified criteria (≥6 for probable AIH and ≥7 for definite AIH). This resulted in similar sensitivity of the updated simplified criteria and the simplified criteria (88% vs. 87%) for probable AIH, but the specificity increased from 80% to 94% (Table 6). For definite AIH, the updated simplified criteria had 57% sensitivity and 97% specificity, while the simplified criteria had 63% sensitivity and 89% specificity.

### Performance of the Four Diagnostic Criteria for Autoimmune Hepatitis

**Figure 2.**
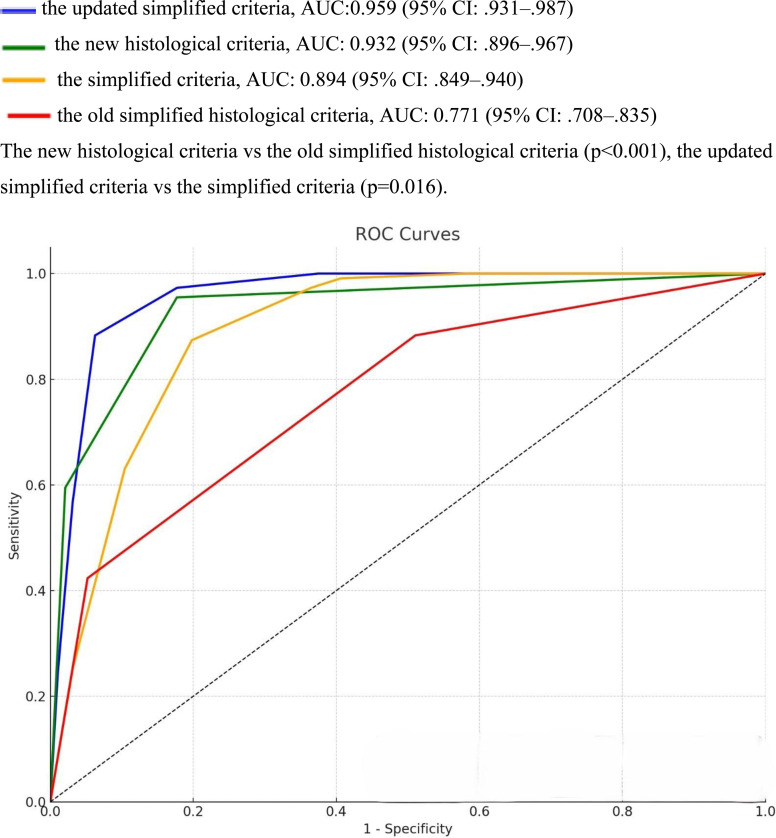
Receiver operating characteristic curves showing the diagnostic performance of 4 diagnostic criteria for autoimmune hepatitis. 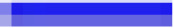
The updated simplified criteria, AUC:0.959 (95% CI: .931-.987). 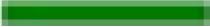
The new histological criteria, AUC: 0.932 (95% CI: .896-.967). 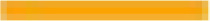
The simplified criteria, AUC: 0.894 (95% CI: .849-.940). 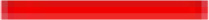
The old simplified histological criteria, AUC: 0.771 (95% CI: .708-.835). The new histological criteria vs the old simplified histological criteria (*P* < .001), the updated simplified criteria vs the simplified criteria (*P* = .016).

Figure 2 shows the performance of the 4 diagnostic AIH criteria. The area under the curve (AUC) was 0.932 (95% CI: .896-.967) for the new HC and 0.771 (95% CI: .708-.835) for the old simplified HC (*P* < .001), 0.959 (95% CI: .931-.987) for the updated simplified criteria and 0.894 (95% CI: .849-.940) for the simplified criteria (*P* = .016).

## Discussion

In this retrospective study from 3 referral centers, the performance of the new HC^9^ and a new updated simplified AIH criteria in patients was evaluated without features of acute liver failure at presentation. The new AIH-PG criteria^9^ clearly demonstrate higher sensitivity and specificity for possible/compatible AIH. The sensitivity of the new HC for likely/typical AIH was not optimal, but still better than the old simplified HC. The sensitivity of the simplified criteria and the new updated simplified criteria was similar and high for probable AIH, but the updated simplified criteria showed a greater specificity for both probable and definitive AIH. The concordance between the AIH-PG HC and updated simplified criteria produced a greater diagnostic ability (AUC: 0.932 and 0.959), consistent with better accuracy than the old simplified HC and simplified criteria (AUC:0.771 and 0.894).

The histological features of the old simplified criteria have not been extensively validated. In 1 study,^7^ the simplified HC had high specificity (91%), but sensitivity was low (40%) for typical AIH. Another study^8^ reported that neither rosettes nor emperipolesis are sensitive and specific histological features of AIH. Moreover, evaluation of rosettes and emperipolesis is suffering from technical difficulties as well as a lack of standardized criteria for assessment.^7^^,^^8^ To overcome these limitations, the international AIH-PG proposed a new HC for AIH diagnosis in 2022.^9^ The performance of the new HC was first evaluated in 47 AIH patients from Italy.^19^ In this study, 95.7% (45/47) of patients reached an AIH diagnosis, with 64% (30/47) of patients classified as “likely” AIH and 32% (15/47) as “possible” AIH. Importantly, 2 (4%) cases classified as “unlikely” AIH had a clinical history suggestive of DILI. In our preliminary study of 78 AIH patients,^18^ the old simplified HC classified 33 (42.3%) patients as compatible with AIH and 36 (46.2%) patients as typical AIH, while the new HC identified 29 (37.2%) as possible AIH, and 46 (59.0%) as likely AIH. These 2 studies supported better sensitivity of the new HC.

The new HC were validated in a large cohort of patients with various liver disorders from China.^10^ When comparing the new HC and the old simplified HC, the sensitivity for a score of ≥1 was 100% vs. 98.6%, and the specificity was 69.0% vs. 54.9%. The sensitivity for a score of ≥2 was 73.6% vs. 42.9%, and the specificity was 100% vs. 98.7%. The new HC showed significantly better diagnostic performance than the old simplified HC. The AUC of the new HC was higher than that of the simplified HC and also the simplified criteria (AUC: 0.959, 0.858, and 0.784). Our present study results are in concordance with the Chinese study [10]. In our study population and in the Chinese study, a score of possible AIH had a sensitivity of 95% vs. 100% and a specificity of 82% vs. 69.0%. For likely AIH, the sensitivity was 60% vs. 73.6% and the specificity was 98% vs. 100%. These results clearly support that the new HC are better than the old HC and can accurately identify AIH.

Autoimmune hepatitis frequently shows an acute onset (absent or low titers of autoantibodies with normal or slightly raised IgG levels), and the performance of the new HC is relevant in such a clinical setting. In our cohort, 35 AIH patients demonstrated an acute presentation. According to the old simplified HC, 63% (22/35) of the patients, and according to the new criteria 74% (26/34) of the patients received the maximum histological score (+2 points), suggesting better performance of the new criteria in this population. In a previous study [19], the new HC identified all 41 acute onset AIH as either “likely” or “possible” AIH. A study from China^10^ included only 13 acute-onset AIH, and the new HC showed better performance than the old HC by upgrading 5 patients from “compatible” to “likely” AIH. This study^10^ also evaluated 107 DILI to assess the performance of the new HC in patients with acute onset liver injury. Of note, the new HC did not perform well in this population as they upgraded the classification of 17 DILI cases by adding +1 point.

Discrimination of AIH from DILI is challenging based on histologic findings. Drug-induced liver injury cases were specifically induced to enhance the value of our study analyses. In our 35 DILI patients, the new HC downgraded 11 cases (2 from typical to possible and 9 from compatible to unlikely) while 4 DILI cases had an upgrade from atypical to possible AIH. Among our DILI patients, 21 met “acute onset” criteria. The old simplified HC considered 10 cases as probable AIH and 2 cases as typical AIH. On the other hand, the new HC ascribed 5 DILI patients as possible AIH and only 1 as likely AIH. Overall, the new HC showed better performance than the simplified HC in the DILI population.

In our cohort, immunofluorescence testing was used for detecting serum ANA, by using HEp-2 cells as substrate. The results of the IAIHG modified cutoffs were incorporated for immunoserological testing^11^ and the new histologic criteria^9^ into the simplified criteria. The sensitivity for the updated simplified criteria and the simplified criteria for probable AIH remained comparable (88% vs. 87%) while the specificity significantly increased from 80% to 94%.

In the first study,^6^ the AIH simplified criteria showed 88% sensitivity and 97% specificity for probable AIH diagnosis, and these criteria were then proposed for clinical use. Subsequent studies from the USA,^20^ the United Kingdom,^21^ and China^22^ also reported very good sensitivity (95%, 90%, and 90%) and specificity (90%, 98%, and 95%) of the simplified criteria in their cohorts. However, a recent large cohort study from China^10^ showed only 58.4% sensitivity and 80.7% specificity of the simplified criteria for a diagnosis of AIH. In our cohort, the specificity of the simplified criteria for probable and definitive AIH diagnosis was 80% and 89%, which are clearly not optimal. Overall, the AUC for the prediction of AIH was 0.784 in the study from China^10^ and was 0.894 in our study. These rates are obviously lower than previous studies that reported 0.934 and 0.977 AUC for the simplified criteria.^21^^,^^22^ These discrepant results between the studies lead to uncertainty about the diagnostic utility of the simplified criteria in general. Therefore, it is thought that the diagnostic approach for AIH needs to be updated in view of current advances, including the present study.

The retrospective nature may be the main limitation of our study. However, histological data were carefully re-evaluated by 2 expert hepato-pathologists, and tissue specimens that contained at least 8 portal areas were included in the study. Our patients were diagnosed when the simplified criteria were routinely used for diagnosing AIH, and only patients with complete laboratory and clinical data pertinent to the simplified criteria were included in this study. All centers used the same manufacturer’s kits for immunoserological assessment.^23^ This minimized discordance between the collected data. Of note, the performance of the new HC in AIH patients presenting with features of acute liver failure could not be evaluated. The incidence of AIH with features of liver failure is low, and obtaining liver tissue that adheres to standardized protocols is difficult in this setting. The diagnostic performance of the new HC should be evaluated by an international multi-center cooperation in this special group of patients. Interobserver agreement among 2 pathologists could not be evaluated. Therefore, information about the ease of use of these criteria cannot be provided for the pathologists.

In conclusion, it was shown that the new HC has high sensitivity and specificity for diagnosing AIH in Turkish patients with liver disease. The simplified criteria could be successfully updated by incorporating the new HC and the modified immunoserological cutoffs. The updated simplified criteria resulted in better diagnostic accuracy. It is suggested that the performance of the updated simplified criteria should be validated in other large cohorts of patients with various liver disorders before they are recommended for implementation in routine clinical use.

## Supplementary Materials

Supplementary Material

## Figures and Tables

**Table 1.** The New 2022 Histological Criteria in the Settings of Both Portal and Lobular Hepatitis

	Portal Hepatitis	Lobular Hepatitis
Likely AIH	Portal lymphoplasmacytic infiltrate Plus 1 or both of the following features: 1. More than mild interface hepatitis 2. More than mild lobular inflammation In the absence of histological features suggestive of another liver disease	More than mild lobular hepatitis (+/− centrilobular necroinflammation) Plus at least one of the following features: 1. Lymphoplasmacytic infiltrates 2. Interface hepatitis 3. Portal-based fibrosis In the absence of histological features suggestive of another liver disease
Possible AIH	Portal lymphoplasmacytic infiltrate without either of the likely features 1 or 2 above in the absence of histological features suggestive of another liver disease OR with 1 or both of the likely features above in the presence of histological features suggestive of another liver disease	Any lobular hepatitis (+/− centrilobular necroinflammation) without any of the likely features 1-3 above in the absence of histological features suggestive of another liver disease OR with any of the likely features above in the presence of histological features suggestive of another liver disease
Unlikely AIH	Portal hepatitis without either of the likely features above in the presence of histological features suggestive of another liver disease	Any lobular hepatitis without any of the likely features above in the presence histological features suggestive of another liver disease

AIH, autoimmune hepatitis.

**Table 2.** Updated Simplified Criteria for Autoimmune Hepatitis

Variables	Cutoff	Points
ANA or SMA	Positive^1^	1
ANA or SMA	Strongly positive^2^	2
LKM	≥1/40	2
SLA/LP	Positive	2
Serum IgG	> Upper limit normal > 1.1 × upper normal limit	1 2
Liver histology	Possible AIH Likely AIH	1 2
Absence of viral hepatitis	Yes	2
≥6: probable AIH ≥7: definite AIH		

Enzyme linked immunoassay (ELISA) with cutoffs validated locally.

Liver histology is based on 2022 histological criteria.

AIH, autoimmune hepatitis; ANA, antinuclear antibodies; IgG, Immunoglobulin G; LKM, liver kidney microsomal type 1 antibody; SLA/LP, soluble liver pancreas antigen; SMA, smooth muscle antibodies.

^1^Immunofluorescence Test (IFT): ≥1:40 when assessed on tissue sections; ≥1:80 or 1:160 for ANA when assessed on HEp2 cells.

^2^IFT: ≥1:80 when assessed on tissue sections; ≥1:160 or 1:320 for ANA when assessed on HEp-2 cells.

**Table 3.** Demographic and Laboratory Features of the Study Population

	AIH (n = 111)	DILI (n = 35)	PBC (n = 33)	MAFLD (n = 28)
Median age (years)	53 (36-68)*	46 (33-57)*	53 (45-62)*	50 (18-70)*
Female (%)	85	63	82	46
ALT (IU/L)	328 (18-2823)*	839 ± 832**	62 ± 36**	101 ± 36**
AST (IU/L)	321 (18-2155)*	694 ± 670**	46 (6-193)*	74 ± 36**
GGT (IU/L)	113 (7-1050)*	222 (98-2150)*	280±267**	71 (47-100)*
ALP (IU/L)	158 (54-925)*	193 (75-1800)*	294 ± 198**	69 ± 21**
Bilirubin (mg/dL)	1.68 (0.2-29)*	11 (1.8-30)*	0.63 (0.6-3)*	0.22 (0.18-0.28)
INR	1.08 ± 0.2**	1.30 (0.89-1.80)*	0.98 ± 0.1**	0.82 ± 1.01**
IgG (mg/L)	1880 ± 1550**	1350 (1130-3700)*	1250 (100-1440)*	1133 ± 233**
ANA (+) [n (%)]	86 (77)	14 (40)	19 (57)	2 (7)
ANA (+) > 1/160 [n(%)]	38 (34)	5 (14)	13 (39)	0 (0)
SMA (+) [n (%)]	22 (20)	1 (3)	1 (3)	0 (0)
Anti-LKM-1 (+) [n (%)]	11 (10)	0 (0)	1 (3)	0 (0)
SLA/LP (+) [n (%)]	6 (5)	0 (0)	0 (0)	0 (0)
AMA (+) [n (%)]	10 (9)	0 (0)	31 (94)	0 (0)

Normal ranges: ALT (0-49 IU/L), AST (0-34 IU/L), ALP (46-116 IU/L), GGT (0-73 IU/L); Bilirubin (0.3-1.2 mg/dl), INR (0.8-1.2); IgG (751-1560 mg/L).

AIH, autoimmune hepatitis; ALP, alkaline phosphatase; ALT, alanine aminotransferase; AMA, anti-mitochondrial antibody; AST, aspartate aminotransferase; DILI, drug-induced liver injury; GGT, gamma-glutamyl transferase; INR, international normalized ratio; MAFLD, metabolic dysfunction–associated fatty liver disease; PBC, primary biliary cirrhosis.

*Median 25%-75%.

**Mean ± SD.

**Table 4.** Histological Features of Study Groups

Histological findings, n (%)	AIH (n = 111)	DILI (n = 35)	PBC (n = 33)	MAFLD (n = 28)
Portal lymphoplasmacytic infiltration	98 (88)	29 (83)	33 (100)	10 (35)
Lobular lymphoplasmacytic infiltration	46 (41)	9 (26)	8 (24)	2 (7)
Interface hepatitis	105 (95)	16 (46)	25 (76)	3 (11)
Rosette formation	76 (68)	8 (23)	1 (3)	0 (0)
Emperipolesis	66 (59)	4 (11)	0 (0)	1 (3)

AIH, autoimmune hepatitis; DILI, drug-induced liver injury; MAFLD, metabolic dysfunction–associated fatty liver disease; PBC, primary biliary cirrhosis.

**Table 5.** Diagnostic Parameters of the Pathological Scoring Systems for Autoimmune Hepatitis

Variables, n (%)	The 2008 Simplified Histological Criteria		The 2022 Histological Criteria	
	≥1	=2	≥1	=2
Sensitivity	88 (98/111)	42 (47/111)	95 (106/111)	60 (66/111)
Specificity	49 (47/96)	95 (91/96)	82 (79/96)	98 (94/96)
Positive predictive value	67 (98/147)	90 (47/52)	86 (106/123)	97 (66/68)
Negative predictive value	78 (47/60)	59 (91/155)	94 (79/84)	68 (94/139)
Accuracy	70 (145/207)	67 (138/207)	89 (185/207)	77 (160/207)
	Compatible AIH = 1, typical AIH = 2		Possible AIH = 1, likely AIH = 2	

AIH, autoimmune hepatitis.

**Table 6.** Performance of the Simplified Criteria and New Updated Simplified Criteria

Variables, n (%)	The Simplified Criteria		The Updated Simplified Criteria	
	≥6	≥7	≥6	≥7
Sensitivity	87 (97/111)	63 (70/111)	88 (98/111)	57 (63/111)
Specificity	80 (77/96)	89 (86/96)	94 (90/96)	97 (93/96)
Positive predictive value	84 (97/116)	88 (70/80)	94 (98/104)	96 (63/66)
Negative predictive value	85 (77/91)	68 (86/127)	87 (90/103)	66 (93/141)
Accuracy	84 (174/207)	75 (156/207)	91 (188/207)	75 (156/207)

## Data Availability

The data that support the findings of this study are presented in the manuscript
