# Supplementary material for: Evaluation of Histological Criteria and Immunoserological Testing of Simplified Criteria for the Diagnosis of Autoimmune Hepatitis
Source: Turk J Gastroenterol. 2025 Sep 18;37(2):223–32. doi: 10.5152/tjg.2025.25402 (PMC12910304; doi:10.5152/tjg.2025.25402)
Supplement: Supplementary Material [file supplementary_material.pdf]

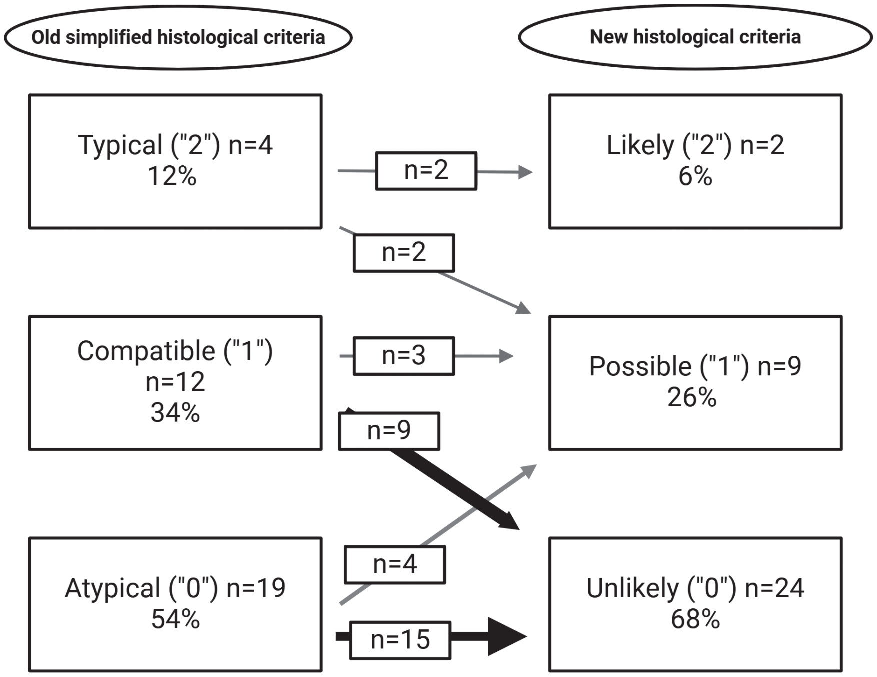

Supplementary Figure 1. Comparison and transitions between the simplified histological criteria and the new histological criteria in the drug induced liver injury group.

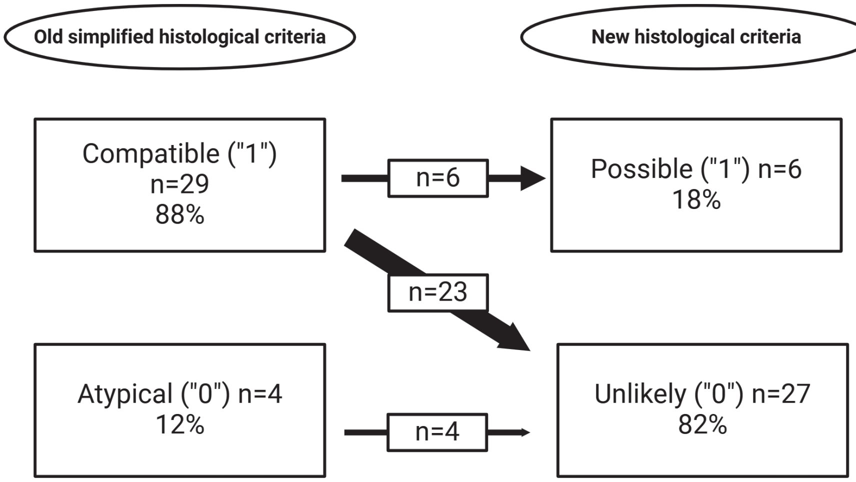

Supplementary Figure 2. Comparison and transitions between the simplified histological criteria and the new histological criteria in the primary biliary cholangitis group.

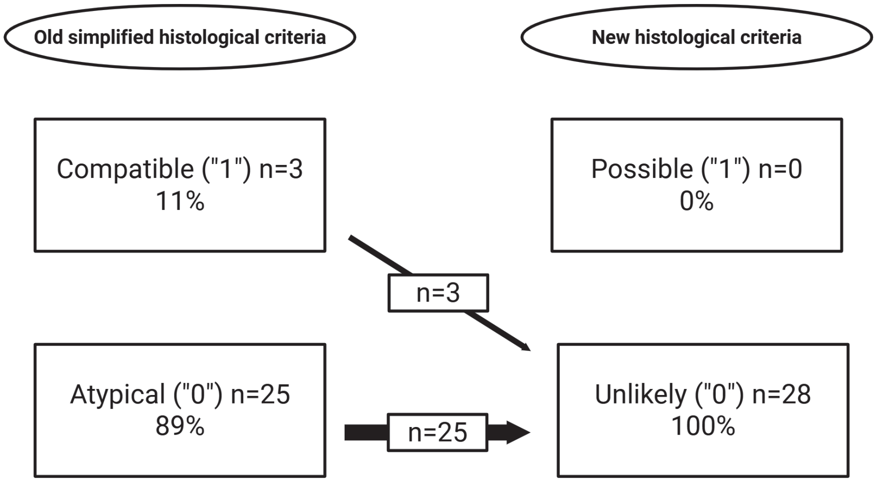

Supplementary Figure 3. Comparison and transitions between the simplified histological criteria and the new histological criteria in the metabolic dysfunction- associated fatty liver disease group.
